# (3*R*,6*R*,12*R*,20*S*,24*S*)-20,24-Ep­oxy­dammarane-3,6,12,25-tetraol dihydrate

**DOI:** 10.1107/S1600536810046362

**Published:** 2010-11-17

**Authors:** Qing-Guo Meng, Lian-Dong Liu, Huan-Mei Guo, Yi Bi, Liang Wang

**Affiliations:** a School of Pharmacy, Yantai University, Yantai 264005, People’s Republic of China; b College of Chemistry, Chemical Engineering and Materials Science, Shandong Normal University, Jinan 250014, People’s Republic of China; c Microscale Science Institute, Weifang University, Weifang 261061, People’s Republic of China

## Abstract

The title compound, C_30_H_52_O_5_·2H_2_O, was degraded from pseudoginsenoside F11 which was extracted and seperated from *Panax quinquefolium saponin*. The three six-membered rings are in chair conformations. The five-membered ring is in an envelope conformation and the tetra­hydro­furan ring has a conformation inter­mediate between half-chair and envelope. In the crystal, inter­molecular O—H⋯O hydrogen bonds link mol­ecules into a three-dimensional network. Intra­molecular O—H⋯O hydrogen bonds also occur.

## Related literature

For background and the medicinal properties of *Panax ginseng* and *Panax quinquefolium*, see: Iljin *et al.* (1982 ▶); Shi *et al.* (1992 ▶); Shibata *et al.* (1985 ▶); Takano *et al.* (1999 ▶); Yu *et al.* (2007 ▶).
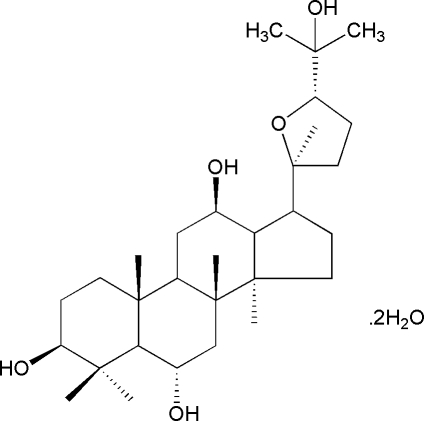


## Experimental

### 

#### Crystal data

C_30_H_52_O_5_·2H_2_O
*M* *_r_* = 528.75
Orthorhombic, 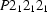

*a* = 11.4575 (15) Å
*b* = 15.457 (2) Å
*c* = 16.726 (2) Å
*V* = 2962.2 (7) Å^3^
*Z* = 4
Mo *K*α radiation
μ = 0.08 mm^−1^
*T* = 298 K
0.51 × 0.38 × 0.32 mm

#### Data collection

Bruker SMART CCD diffractometer
15595 measured reflections
3095 independent reflections
2768 reflections with *I* > 2σ(*I*)
*R*_int_ = 0.032

#### Refinement

*R*[*F* ^2^ > 2σ(*F* ^2^)] = 0.042
*wR*(*F* ^2^) = 0.108
*S* = 1.05
3095 reflections
346 parameters
6 restraints
H-atom parameters constrained
Δρ_max_ = 0.18 e Å^−3^
Δρ_min_ = −0.16 e Å^−3^

### 

Data collection: *SMART* (Bruker, 1997 ▶); cell refinement: *SAINT* (Bruker, 1997 ▶); data reduction: *SAINT*; program(s) used to solve structure: *SHELXS97* (Sheldrick, 2008 ▶); program(s) used to refine structure: *SHELXL97* (Sheldrick, 2008 ▶); molecular graphics: *SHELXTL* (Sheldrick, 2008 ▶); software used to prepare material for publication: *SHELXTL*.

## Supplementary Material

Crystal structure: contains datablocks global, I. DOI: 10.1107/S1600536810046362/lh5154sup1.cif

Structure factors: contains datablocks I. DOI: 10.1107/S1600536810046362/lh5154Isup2.hkl

Additional supplementary materials:  crystallographic information; 3D view; checkCIF report

## Figures and Tables

**Table 1:** Hydrogen-bond geometry (Å, °)

*D*—H⋯*A*	*D*—H	H⋯*A*	*D*⋯*A*	*D*—H⋯*A*
O7—H7*B*⋯O4	0.84	2.05	2.836 (3)	155
O6—H6*C*⋯O5^i^	0.83	2.24	2.791 (3)	124
O7—H7*A*⋯O1^ii^	0.84	2.02	2.829 (3)	160
O6—H6*D*⋯O3^iii^	0.85	2.04	2.879 (3)	167
O5—H5⋯O7^iv^	0.82	1.92	2.734 (3)	174
O4—H4⋯O6^v^	0.82	2.00	2.795 (3)	164
O3—H3⋯O2	0.82	1.82	2.627 (2)	170
O1—H1⋯O3	0.82	2.14	2.938 (3)	164

Symmetry codes: (i) 
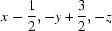
; (ii) 
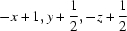
; (iii) 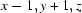; (iv) 
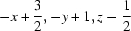
; (v) 
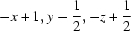
.

**Fig. 1.**
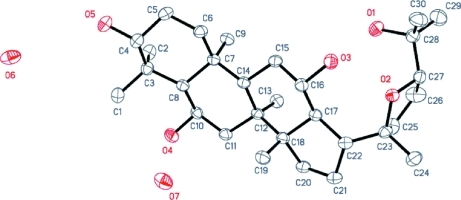
The molecular structure of the title compound with the atom-labeling scheme. Displacement ellipsoids are drawn at the 30% probability level. H atoms are not shown.

## supplementary crystallographic information

### Comment

Both Panax ginseng and Panax quinquefolium, belonging to Araliaceae, are well known traditional medicinal herbs. They are used as tonics and for the treatment for diseases, such as tumor and myocardial ischemia. Panax ginseng contains a number of saponins, namely ginsenoside and an oleanolic acid-type saponin in addition to the major protopanaxadiol and protopanaxatriol-type saponins (Shibata *et al.*,1985). Panax quinquefolium contains an ocotillol-type (20*S*, 24*R*-epoxyside) saponin with high anti-tumor activity (Takano *et al.*,1999), as well as oleanolic acid-type saponin, protopanaxadiol and protopanaxatriol-type saponins. (3*R*,6*R*,12*R*,20S,24*S*)-20,24-epoxy-dammarane-3,6,12,25-tetraol is found to possess cardioprotective effects on myocardial injury induced by isoproterenol in rats (Yu *et al.*,2007). As part of our ongoing investigation of ocotillol-type compounds and their cardioprotective effect on myocardial injury, we report here the crystal structure of the title compound, (I).

The molecular structure of (I) is shown in Fig. 1. In the molecule, all bond lengths and angles are within normal ranges (Shi *et al.*,1992; Iljin *et al.*,1982). The six-membered rings A(C3/C4/C5/C6/C7/C8), B(C7/C8/C10/C11/C12/C14), and C(C12/C14/C15/C16/C17/C18) are in chair conformations that depart significantly from ideal geometry to accommodate for intramolecular non-bonded 1,3-diaxial interactions involving the methyl groups. The five-membered ring D(C13/C14/C15/C16/C17) has an envelope form with C18 as the out-of-plane atom. The tetrahydrofuran ring has a conformation intermediate between half-chair and envelope forms. In the crystal structure intermolecular O-H···O hydrogen bonds link molecules into a three-dimensional network.

### Experimental

Pseudoginsenoside F11 was extracted and seperated from Panax quinquefolium saponin. (3*R*,6*R*,12*R*,20S,24*S*)-20,24-epoxy-dammarane-3,6,12,25-tetraol was degraded from Pseudoginsenoside F11 with sodium in glycerine and seperated by flash chromatography. Finally, the crystals were dried at room temperature. Single crystals of compound (I) suitable for X-ray measurements were obtained by recrystallization from ethyl acetate at room temperature.

### Refinement

H atoms bonded to C atoms were fixed geometrically and allowed to ride on their attached atoms, with C—H distances in the range 0.96–0.98 Å, and with *U*_iso_(H) = 1.2*U*_eq_(C) or 1.5*U*_eq_(C_methyl_). The H atoms bonded to hydroxy O atoms were included with O-H = 0.82Å and *U*_iso_(H) = 1.5*U*_eq_(O) and water H atoms were included in 'as-found' positions with *U*_iso_(H) = 0.12Å^3^. In the absence of anomlaous dispersion effects Friedel pairs were merged. The absolute configuration is based on unchanging stereochemical centers in the synthesis.

### Crystal data

**Table:**

C_30_H_52_O_5_·2H_2_O	*F*(000) = 1168
*M**_r_* = 528.75	*D*_x_ = 1.186 Mg m^−^^3^
Orthorhombic, *P*2_1_2_1_2_1_	Mo *K*α radiation, λ = 0.71073 Å
Hall symbol: P 2ac 2ab	Cell parameters from 5972 reflections
*a* = 11.4575 (15) Å	θ = 2.4–28.0°
*b* = 15.457 (2) Å	µ = 0.08 mm^−^^1^
*c* = 16.726 (2) Å	*T* = 298 K
*V* = 2962.2 (7) Å^3^	Block, colourless
*Z* = 4	0.51 × 0.38 × 0.32 mm

### Data collection

**Table:**

Bruker SMART CCD diffractometer	2768 reflections with *I* > 2σ(*I*)
Radiation source: fine-focus sealed tube	*R*_int_ = 0.032
graphite	θ_max_ = 25.5°, θ_min_ = 2.2°
φ and ω scans	*h* = −13→13
15595 measured reflections	*k* = −18→18
3095 independent reflections	*l* = −10→20

### Refinement

**Table:**

Refinement on *F*^2^	Primary atom site location: structure-invariant direct methods
Least-squares matrix: full	Secondary atom site location: difference Fourier map
*R*[*F*^2^ > 2σ(*F*^2^)] = 0.042	Hydrogen site location: inferred from neighbouring sites
*wR*(*F*^2^) = 0.108	H-atom parameters constrained
*S* = 1.05	*w* = 1/[σ^2^(*F*_o_^2^) + (0.0592*P*)^2^ + 0.5608*P*] where *P* = (*F*_o_^2^ + 2*F*_c_^2^)/3
3095 reflections	(Δ/σ)_max_ < 0.001
346 parameters	Δρ_max_ = 0.18 e Å^−^^3^
6 restraints	Δρ_min_ = −0.16 e Å^−^^3^

### Special details

**Table:**

Geometry. All e.s.d.'s (except the e.s.d. in the dihedral angle between two l.s. planes) are estimated using the full covariance matrix. The cell e.s.d.'s are taken into account individually in the estimation of e.s.d.'s in distances, angles and torsion angles; correlations between e.s.d.'s in cell parameters are only used when they are defined by crystal symmetry. An approximate (isotropic) treatment of cell e.s.d.'s is used for estimating e.s.d.'s involving l.s. planes.
Refinement. Refinement of *F*^2^ against ALL reflections. The weighted *R*-factor *wR* and goodness of fit *S* are based on *F*^2^, conventional *R*-factors *R* are based on *F*, with *F* set to zero for negative *F*^2^. The threshold expression of *F*^2^ > σ(*F*^2^) is used only for calculating *R*-factors(gt) *etc*. and is not relevant to the choice of reflections for refinement. *R*-factors based on *F*^2^ are statistically about twice as large as those based on *F*, and *R*- factors based on ALL data will be even larger.

### Fractional atomic coordinates and isotropic or equivalent isotropic displacement parameters (Å^2^)

**Table:**

	*x*	*y*	*z*	*U*_iso_*/*U*_eq_	
C1	0.7657 (3)	0.50304 (19)	0.18423 (19)	0.0600 (8)	
H1A	0.7841	0.5541	0.1541	0.090*	
H1B	0.8326	0.4859	0.2149	0.090*	
H1C	0.7017	0.5149	0.2196	0.090*	
C2	0.6080 (2)	0.44716 (19)	0.09599 (19)	0.0546 (7)	
H2A	0.6027	0.5058	0.0774	0.082*	
H2B	0.5531	0.4384	0.1386	0.082*	
H2C	0.5904	0.4083	0.0528	0.082*	
C3	0.7318 (2)	0.42959 (16)	0.12652 (17)	0.0436 (6)	
C4	0.8223 (2)	0.43365 (17)	0.05835 (17)	0.0453 (6)	
H4A	0.8999	0.4327	0.0831	0.054*	
C5	0.8168 (3)	0.35892 (18)	0.00041 (16)	0.0485 (7)	
H5A	0.8769	0.3658	−0.0400	0.058*	
H5B	0.7417	0.3588	−0.0262	0.058*	
C6	0.8343 (3)	0.27323 (17)	0.04358 (15)	0.0437 (6)	
H6A	0.9126	0.2716	0.0654	0.052*	
H6B	0.8276	0.2265	0.0051	0.052*	
C7	0.7463 (2)	0.25757 (16)	0.11176 (14)	0.0367 (5)	
C8	0.7455 (2)	0.33919 (15)	0.16833 (15)	0.0370 (5)	
H8	0.8237	0.3405	0.1920	0.044*	
C9	0.6270 (2)	0.23805 (18)	0.07120 (16)	0.0452 (6)	
H9A	0.6200	0.2718	0.0232	0.068*	
H9B	0.5648	0.2527	0.1072	0.068*	
H9C	0.6227	0.1777	0.0581	0.068*	
C10	0.6633 (2)	0.32106 (15)	0.23917 (16)	0.0402 (6)	
H10	0.5864	0.3064	0.2173	0.048*	
C11	0.7058 (2)	0.24394 (16)	0.28745 (15)	0.0431 (6)	
H11A	0.6510	0.2339	0.3307	0.052*	
H11B	0.7802	0.2589	0.3114	0.052*	
C12	0.7209 (2)	0.15891 (15)	0.24068 (14)	0.0363 (5)	
C13	0.5974 (2)	0.12287 (18)	0.22259 (17)	0.0451 (6)	
H13A	0.5619	0.1035	0.2714	0.068*	
H13B	0.6036	0.0752	0.1860	0.068*	
H13C	0.5504	0.1676	0.1992	0.068*	
C14	0.7921 (2)	0.17999 (15)	0.16346 (15)	0.0359 (5)	
H14	0.8678	0.2000	0.1836	0.043*	
C15	0.8217 (2)	0.09726 (16)	0.11639 (15)	0.0416 (6)	
H15A	0.7500	0.0713	0.0970	0.050*	
H15B	0.8690	0.1124	0.0704	0.050*	
C16	0.8869 (2)	0.03177 (16)	0.16698 (15)	0.0397 (6)	
H16	0.9597	0.0583	0.1856	0.048*	
C17	0.8132 (2)	0.00865 (15)	0.23932 (14)	0.0367 (5)	
H17	0.7367	−0.0091	0.2190	0.044*	
C18	0.7928 (2)	0.09115 (16)	0.29063 (15)	0.0404 (6)	
C19	0.9103 (3)	0.12856 (18)	0.32081 (17)	0.0514 (7)	
H19A	0.8969	0.1624	0.3681	0.077*	
H19B	0.9439	0.1645	0.2801	0.077*	
H19C	0.9628	0.0820	0.3330	0.077*	
C20	0.7324 (3)	0.05094 (18)	0.36378 (15)	0.0518 (7)	
H20A	0.7330	0.0905	0.4088	0.062*	
H20B	0.6523	0.0353	0.3516	0.062*	
C21	0.8061 (3)	−0.02966 (19)	0.38146 (16)	0.0542 (7)	
H21A	0.7589	−0.0739	0.4068	0.065*	
H21B	0.8705	−0.0153	0.4167	0.065*	
C22	0.8527 (2)	−0.06215 (16)	0.29903 (15)	0.0414 (6)	
H22	0.9382	−0.0629	0.3008	0.050*	
C23	0.8091 (2)	−0.15422 (16)	0.28113 (15)	0.0406 (6)	
C24	0.8690 (3)	−0.2194 (2)	0.33484 (18)	0.0605 (8)	
H24A	0.8404	−0.2763	0.3230	0.091*	
H24B	0.8529	−0.2057	0.3897	0.091*	
H24C	0.9517	−0.2174	0.3258	0.091*	
C25	0.6758 (2)	−0.16592 (19)	0.28239 (19)	0.0530 (7)	
H25A	0.6509	−0.1916	0.3325	0.064*	
H25B	0.6368	−0.1106	0.2759	0.064*	
C26	0.6484 (3)	−0.2245 (3)	0.2139 (2)	0.0717 (10)	
H26A	0.5886	−0.1991	0.1803	0.086*	
H26B	0.6202	−0.2798	0.2334	0.086*	
C27	0.7600 (2)	−0.23660 (17)	0.16702 (16)	0.0441 (6)	
H27	0.7902	−0.2948	0.1777	0.053*	
C28	0.7516 (3)	−0.22421 (18)	0.07639 (16)	0.0462 (6)	
C29	0.6604 (3)	−0.2842 (2)	0.0421 (2)	0.0706 (9)	
H29A	0.5848	−0.2681	0.0619	0.106*	
H29B	0.6775	−0.3426	0.0577	0.106*	
H29C	0.6609	−0.2800	−0.0152	0.106*	
C30	0.8687 (3)	−0.2400 (2)	0.03777 (18)	0.0629 (9)	
H30A	0.8627	−0.2304	−0.0188	0.094*	
H30B	0.8925	−0.2986	0.0475	0.094*	
H30C	0.9253	−0.2011	0.0600	0.094*	
O1	0.71201 (17)	−0.13798 (12)	0.05889 (13)	0.0524 (5)	
H1	0.7640	−0.1033	0.0693	0.079*	
O2	0.84059 (14)	−0.17448 (10)	0.19926 (10)	0.0380 (4)	
O3	0.91541 (18)	−0.04074 (11)	0.11696 (11)	0.0491 (5)	
H3	0.8984	−0.0858	0.1401	0.074*	
O4	0.64852 (19)	0.39272 (12)	0.29256 (12)	0.0552 (5)	
H4	0.7100	0.4020	0.3161	0.083*	
O5	0.81123 (18)	0.51420 (12)	0.01735 (13)	0.0559 (5)	
H5	0.8679	0.5214	−0.0118	0.084*	
O6	0.1664 (2)	0.9510 (2)	0.11289 (16)	0.1016 (10)	
H6D	0.0926	0.9519	0.1058	0.122*	
H7A	0.4471	0.4180	0.4365	0.122*	
H6C	0.1989	0.9933	0.0921	0.122*	
H7B	0.5268	0.4294	0.3756	0.122*	
O7	0.5037 (2)	0.44817 (18)	0.42014 (16)	0.0821 (8)	

### Atomic displacement parameters (Å^2^)

**Table:**

	*U*^11^	*U*^22^	*U*^33^	*U*^12^	*U*^13^	*U*^23^
C1	0.077 (2)	0.0443 (14)	0.0584 (17)	−0.0088 (15)	0.0047 (17)	0.0012 (14)
C2	0.0428 (15)	0.0536 (16)	0.0674 (19)	0.0066 (13)	0.0042 (14)	0.0095 (15)
C3	0.0435 (14)	0.0405 (13)	0.0469 (15)	0.0016 (11)	0.0028 (12)	0.0015 (12)
C4	0.0392 (14)	0.0479 (14)	0.0489 (15)	−0.0018 (11)	−0.0019 (12)	0.0084 (12)
C5	0.0513 (16)	0.0557 (15)	0.0385 (14)	0.0009 (13)	0.0068 (12)	0.0082 (12)
C6	0.0470 (15)	0.0483 (14)	0.0359 (13)	0.0034 (12)	0.0053 (12)	−0.0002 (11)
C7	0.0348 (12)	0.0427 (13)	0.0326 (12)	−0.0015 (10)	−0.0013 (11)	−0.0019 (10)
C8	0.0348 (12)	0.0408 (13)	0.0354 (12)	−0.0015 (10)	−0.0010 (11)	−0.0029 (11)
C9	0.0441 (15)	0.0518 (15)	0.0399 (14)	−0.0022 (12)	−0.0043 (12)	−0.0057 (13)
C10	0.0409 (13)	0.0398 (12)	0.0398 (14)	−0.0012 (11)	0.0048 (12)	−0.0081 (11)
C11	0.0495 (15)	0.0466 (14)	0.0334 (13)	−0.0027 (12)	0.0083 (12)	−0.0058 (11)
C12	0.0376 (13)	0.0400 (12)	0.0312 (12)	0.0008 (11)	0.0044 (10)	−0.0047 (10)
C13	0.0391 (13)	0.0484 (14)	0.0477 (15)	−0.0028 (11)	0.0068 (12)	−0.0014 (12)
C14	0.0329 (12)	0.0419 (13)	0.0329 (12)	−0.0004 (10)	0.0018 (10)	−0.0022 (10)
C15	0.0482 (15)	0.0442 (13)	0.0325 (12)	0.0013 (11)	0.0102 (12)	−0.0013 (11)
C16	0.0392 (13)	0.0406 (13)	0.0392 (13)	−0.0002 (10)	0.0073 (11)	−0.0032 (11)
C17	0.0353 (12)	0.0402 (12)	0.0346 (12)	−0.0016 (10)	0.0010 (10)	−0.0024 (10)
C18	0.0450 (13)	0.0440 (13)	0.0322 (12)	−0.0006 (11)	0.0014 (11)	−0.0038 (11)
C19	0.0578 (16)	0.0504 (15)	0.0462 (16)	−0.0017 (13)	−0.0124 (14)	−0.0044 (13)
C20	0.0724 (19)	0.0519 (15)	0.0312 (13)	0.0021 (14)	0.0074 (13)	−0.0040 (12)
C21	0.072 (2)	0.0576 (16)	0.0334 (13)	0.0012 (15)	0.0019 (14)	0.0001 (13)
C22	0.0397 (13)	0.0500 (14)	0.0345 (12)	−0.0008 (11)	−0.0013 (11)	0.0006 (11)
C23	0.0420 (13)	0.0451 (13)	0.0349 (13)	0.0025 (11)	0.0070 (11)	0.0045 (11)
C24	0.077 (2)	0.0574 (17)	0.0470 (16)	0.0101 (16)	−0.0033 (16)	0.0092 (15)
C25	0.0437 (15)	0.0515 (15)	0.0639 (19)	−0.0012 (13)	0.0115 (14)	0.0044 (15)
C26	0.0505 (17)	0.106 (3)	0.0582 (19)	−0.0272 (18)	0.0041 (16)	0.001 (2)
C27	0.0484 (15)	0.0363 (12)	0.0478 (15)	−0.0050 (11)	−0.0073 (13)	0.0052 (11)
C28	0.0482 (15)	0.0462 (14)	0.0442 (14)	0.0077 (12)	−0.0062 (13)	0.0008 (12)
C29	0.075 (2)	0.070 (2)	0.067 (2)	−0.0067 (18)	−0.0228 (19)	−0.0056 (18)
C30	0.068 (2)	0.075 (2)	0.0459 (16)	0.0217 (17)	0.0027 (15)	−0.0087 (16)
O1	0.0506 (11)	0.0523 (11)	0.0543 (11)	0.0097 (9)	−0.0071 (10)	0.0098 (10)
O2	0.0353 (8)	0.0418 (9)	0.0370 (9)	0.0006 (7)	0.0022 (8)	0.0003 (8)
O3	0.0587 (11)	0.0409 (9)	0.0476 (10)	0.0016 (9)	0.0195 (10)	−0.0005 (9)
O4	0.0682 (13)	0.0476 (10)	0.0500 (12)	0.0035 (10)	0.0078 (10)	−0.0130 (9)
O5	0.0558 (13)	0.0523 (11)	0.0595 (12)	−0.0010 (9)	0.0062 (10)	0.0165 (10)
O6	0.0586 (15)	0.175 (3)	0.0714 (16)	0.0095 (18)	0.0082 (13)	0.021 (2)
O7	0.0564 (13)	0.1091 (19)	0.0807 (16)	−0.0227 (13)	0.0062 (12)	−0.0208 (16)

### Geometric parameters (Å, °)

**Table:**

C1—C3	1.540 (4)	C17—C18	1.555 (3)
C1—H1A	0.9600	C17—H17	0.9800
C1—H1B	0.9600	C18—C20	1.537 (4)
C1—H1C	0.9600	C18—C19	1.549 (4)
C2—C3	1.532 (4)	C19—H19A	0.9600
C2—H2A	0.9600	C19—H19B	0.9600
C2—H2B	0.9600	C19—H19C	0.9600
C2—H2C	0.9600	C20—C21	1.534 (4)
C3—C4	1.543 (4)	C20—H20A	0.9700
C3—C8	1.570 (3)	C20—H20B	0.9700
C4—O5	1.427 (3)	C21—C22	1.561 (4)
C4—C5	1.509 (4)	C21—H21A	0.9700
C4—H4A	0.9800	C21—H21B	0.9700
C5—C6	1.522 (4)	C22—C23	1.538 (4)
C5—H5A	0.9700	C22—H22	0.9800
C5—H5B	0.9700	C23—O2	1.450 (3)
C6—C7	1.541 (3)	C23—C24	1.514 (4)
C6—H6A	0.9700	C23—C25	1.538 (4)
C6—H6B	0.9700	C24—H24A	0.9600
C7—C9	1.555 (3)	C24—H24B	0.9600
C7—C14	1.569 (3)	C24—H24C	0.9600
C7—C8	1.577 (3)	C25—C26	1.494 (5)
C8—C10	1.539 (3)	C25—H25A	0.9700
C8—H8	0.9800	C25—H25B	0.9700
C9—H9A	0.9600	C26—C27	1.511 (4)
C9—H9B	0.9600	C26—H26A	0.9700
C9—H9C	0.9600	C26—H26B	0.9700
C10—O4	1.433 (3)	C27—O2	1.437 (3)
C10—C11	1.520 (4)	C27—C28	1.531 (4)
C10—H10	0.9800	C27—H27	0.9800
C11—C12	1.539 (3)	C28—O1	1.438 (3)
C11—H11A	0.9700	C28—C30	1.509 (4)
C11—H11B	0.9700	C28—C29	1.511 (4)
C12—C13	1.550 (4)	C29—H29A	0.9600
C12—C14	1.562 (3)	C29—H29B	0.9600
C12—C18	1.573 (4)	C29—H29C	0.9600
C13—H13A	0.9600	C30—H30A	0.9600
C13—H13B	0.9600	C30—H30B	0.9600
C13—H13C	0.9600	C30—H30C	0.9600
C14—C15	1.539 (3)	O1—H1	0.8200
C14—H14	0.9800	O3—H3	0.8200
C15—C16	1.516 (4)	O4—H4	0.8200
C15—H15A	0.9700	O5—H5	0.8200
C15—H15B	0.9700	O6—H6D	0.8537
C16—O3	1.436 (3)	O6—H6C	0.8296
C16—C17	1.518 (3)	O7—H7A	0.8443
C16—H16	0.9800	O7—H7B	0.8426
C17—C22	1.549 (3)		
			
C3—C1—H1A	109.5	C15—C16—H16	108.5
C3—C1—H1B	109.5	C17—C16—H16	108.5
H1A—C1—H1B	109.5	C16—C17—C22	121.2 (2)
C3—C1—H1C	109.5	C16—C17—C18	109.29 (19)
H1A—C1—H1C	109.5	C22—C17—C18	105.51 (18)
H1B—C1—H1C	109.5	C16—C17—H17	106.7
C3—C2—H2A	109.5	C22—C17—H17	106.7
C3—C2—H2B	109.5	C18—C17—H17	106.7
H2A—C2—H2B	109.5	C20—C18—C19	106.4 (2)
C3—C2—H2C	109.5	C20—C18—C17	100.10 (19)
H2A—C2—H2C	109.5	C19—C18—C17	110.8 (2)
H2B—C2—H2C	109.5	C20—C18—C12	117.1 (2)
C2—C3—C1	108.2 (2)	C19—C18—C12	112.3 (2)
C2—C3—C4	111.7 (2)	C17—C18—C12	109.37 (19)
C1—C3—C4	105.3 (2)	C18—C19—H19A	109.5
C2—C3—C8	113.5 (2)	C18—C19—H19B	109.5
C1—C3—C8	110.6 (2)	H19A—C19—H19B	109.5
C4—C3—C8	107.3 (2)	C18—C19—H19C	109.5
O5—C4—C5	110.8 (2)	H19A—C19—H19C	109.5
O5—C4—C3	109.3 (2)	H19B—C19—H19C	109.5
C5—C4—C3	114.5 (2)	C21—C20—C18	103.5 (2)
O5—C4—H4A	107.3	C21—C20—H20A	111.1
C5—C4—H4A	107.3	C18—C20—H20A	111.1
C3—C4—H4A	107.3	C21—C20—H20B	111.1
C4—C5—C6	110.9 (2)	C18—C20—H20B	111.1
C4—C5—H5A	109.5	H20A—C20—H20B	109.0
C6—C5—H5A	109.5	C20—C21—C22	106.2 (2)
C4—C5—H5B	109.5	C20—C21—H21A	110.5
C6—C5—H5B	109.5	C22—C21—H21A	110.5
H5A—C5—H5B	108.1	C20—C21—H21B	110.5
C5—C6—C7	113.7 (2)	C22—C21—H21B	110.5
C5—C6—H6A	108.8	H21A—C21—H21B	108.7
C7—C6—H6A	108.8	C23—C22—C17	115.7 (2)
C5—C6—H6B	108.8	C23—C22—C21	111.0 (2)
C7—C6—H6B	108.8	C17—C22—C21	104.0 (2)
H6A—C6—H6B	107.7	C23—C22—H22	108.6
C6—C7—C9	106.4 (2)	C17—C22—H22	108.6
C6—C7—C14	108.0 (2)	C21—C22—H22	108.6
C9—C7—C14	112.7 (2)	O2—C23—C24	107.7 (2)
C6—C7—C8	108.8 (2)	O2—C23—C22	107.6 (2)
C9—C7—C8	114.3 (2)	C24—C23—C22	110.7 (2)
C14—C7—C8	106.43 (19)	O2—C23—C25	103.6 (2)
C10—C8—C3	116.3 (2)	C24—C23—C25	111.3 (2)
C10—C8—C7	108.65 (19)	C22—C23—C25	115.4 (2)
C3—C8—C7	116.4 (2)	C23—C24—H24A	109.5
C10—C8—H8	104.7	C23—C24—H24B	109.5
C3—C8—H8	104.7	H24A—C24—H24B	109.5
C7—C8—H8	104.7	C23—C24—H24C	109.5
C7—C9—H9A	109.5	H24A—C24—H24C	109.5
C7—C9—H9B	109.5	H24B—C24—H24C	109.5
H9A—C9—H9B	109.5	C26—C25—C23	105.6 (2)
C7—C9—H9C	109.5	C26—C25—H25A	110.6
H9A—C9—H9C	109.5	C23—C25—H25A	110.6
H9B—C9—H9C	109.5	C26—C25—H25B	110.6
O4—C10—C11	108.2 (2)	C23—C25—H25B	110.6
O4—C10—C8	114.3 (2)	H25A—C25—H25B	108.7
C11—C10—C8	110.8 (2)	C25—C26—C27	107.1 (2)
O4—C10—H10	107.8	C25—C26—H26A	110.3
C11—C10—H10	107.8	C27—C26—H26A	110.3
C8—C10—H10	107.8	C25—C26—H26B	110.3
C10—C11—C12	115.8 (2)	C27—C26—H26B	110.3
C10—C11—H11A	108.3	H26A—C26—H26B	108.5
C12—C11—H11A	108.3	O2—C27—C26	105.5 (2)
C10—C11—H11B	108.3	O2—C27—C28	109.2 (2)
C12—C11—H11B	108.3	C26—C27—C28	116.4 (3)
H11A—C11—H11B	107.4	O2—C27—H27	108.5
C11—C12—C13	107.6 (2)	C26—C27—H27	108.5
C11—C12—C14	107.51 (19)	C28—C27—H27	108.5
C13—C12—C14	113.0 (2)	O1—C28—C30	110.1 (2)
C11—C12—C18	111.0 (2)	O1—C28—C29	105.9 (2)
C13—C12—C18	110.0 (2)	C30—C28—C29	110.6 (3)
C14—C12—C18	107.74 (19)	O1—C28—C27	109.7 (2)
C12—C13—H13A	109.5	C30—C28—C27	110.4 (2)
C12—C13—H13B	109.5	C29—C28—C27	110.1 (3)
H13A—C13—H13B	109.5	C28—C29—H29A	109.5
C12—C13—H13C	109.5	C28—C29—H29B	109.5
H13A—C13—H13C	109.5	H29A—C29—H29B	109.5
H13B—C13—H13C	109.5	C28—C29—H29C	109.5
C15—C14—C12	111.37 (19)	H29A—C29—H29C	109.5
C15—C14—C7	115.3 (2)	H29B—C29—H29C	109.5
C12—C14—C7	116.13 (19)	C28—C30—H30A	109.5
C15—C14—H14	104.1	C28—C30—H30B	109.5
C12—C14—H14	104.1	H30A—C30—H30B	109.5
C7—C14—H14	104.1	C28—C30—H30C	109.5
C16—C15—C14	112.2 (2)	H30A—C30—H30C	109.5
C16—C15—H15A	109.2	H30B—C30—H30C	109.5
C14—C15—H15A	109.2	C28—O1—H1	109.5
C16—C15—H15B	109.2	C27—O2—C23	109.80 (19)
C14—C15—H15B	109.2	C16—O3—H3	109.5
H15A—C15—H15B	107.9	C10—O4—H4	109.5
O3—C16—C15	107.9 (2)	C4—O5—H5	109.5
O3—C16—C17	114.01 (19)	H6D—O6—H6C	111.9
C15—C16—C17	109.1 (2)	H7A—O7—H7B	109.7
O3—C16—H16	108.5		
			
C2—C3—C4—O5	53.7 (3)	O3—C16—C17—C18	177.5 (2)
C1—C3—C4—O5	−63.5 (3)	C15—C16—C17—C18	−61.8 (3)
C8—C3—C4—O5	178.7 (2)	C16—C17—C18—C20	−173.0 (2)
C2—C3—C4—C5	−71.3 (3)	C22—C17—C18—C20	−41.2 (3)
C1—C3—C4—C5	171.5 (2)	C16—C17—C18—C19	−60.9 (3)
C8—C3—C4—C5	53.7 (3)	C22—C17—C18—C19	70.9 (2)
O5—C4—C5—C6	177.4 (2)	C16—C17—C18—C12	63.4 (3)
C3—C4—C5—C6	−58.4 (3)	C22—C17—C18—C12	−164.77 (19)
C4—C5—C6—C7	56.6 (3)	C11—C12—C18—C20	70.8 (3)
C5—C6—C7—C9	72.3 (3)	C13—C12—C18—C20	−48.2 (3)
C5—C6—C7—C14	−166.4 (2)	C14—C12—C18—C20	−171.7 (2)
C5—C6—C7—C8	−51.3 (3)	C11—C12—C18—C19	−52.8 (3)
C2—C3—C8—C10	−56.4 (3)	C13—C12—C18—C19	−171.8 (2)
C1—C3—C8—C10	65.4 (3)	C14—C12—C18—C19	64.6 (2)
C4—C3—C8—C10	179.7 (2)	C11—C12—C18—C17	−176.3 (2)
C2—C3—C8—C7	73.7 (3)	C13—C12—C18—C17	64.7 (2)
C1—C3—C8—C7	−164.5 (2)	C14—C12—C18—C17	−58.9 (2)
C4—C3—C8—C7	−50.2 (3)	C19—C18—C20—C21	−71.0 (3)
C6—C7—C8—C10	−176.5 (2)	C17—C18—C20—C21	44.4 (3)
C9—C7—C8—C10	64.7 (3)	C12—C18—C20—C21	162.5 (2)
C14—C7—C8—C10	−60.4 (2)	C18—C20—C21—C22	−32.0 (3)
C6—C7—C8—C3	49.8 (3)	C16—C17—C22—C23	−91.4 (3)
C9—C7—C8—C3	−69.0 (3)	C18—C17—C22—C23	143.9 (2)
C14—C7—C8—C3	165.9 (2)	C16—C17—C22—C21	146.6 (2)
C3—C8—C10—O4	−42.3 (3)	C18—C17—C22—C21	21.9 (3)
C7—C8—C10—O4	−176.0 (2)	C20—C21—C22—C23	−119.1 (3)
C3—C8—C10—C11	−164.9 (2)	C20—C21—C22—C17	6.0 (3)
C7—C8—C10—C11	61.4 (3)	C17—C22—C23—O2	53.1 (3)
O4—C10—C11—C12	177.1 (2)	C21—C22—C23—O2	171.4 (2)
C8—C10—C11—C12	−56.9 (3)	C17—C22—C23—C24	170.6 (2)
C10—C11—C12—C13	−73.3 (3)	C21—C22—C23—C24	−71.2 (3)
C10—C11—C12—C14	48.7 (3)	C17—C22—C23—C25	−61.9 (3)
C10—C11—C12—C18	166.3 (2)	C21—C22—C23—C25	56.3 (3)
C11—C12—C14—C15	174.7 (2)	O2—C23—C25—C26	22.3 (3)
C13—C12—C14—C15	−66.7 (3)	C24—C23—C25—C26	−93.1 (3)
C18—C12—C14—C15	55.1 (3)	C22—C23—C25—C26	139.7 (3)
C11—C12—C14—C7	−50.7 (3)	C23—C25—C26—C27	−7.6 (4)
C13—C12—C14—C7	68.0 (3)	C25—C26—C27—O2	−10.2 (3)
C18—C12—C14—C7	−170.30 (19)	C25—C26—C27—C28	−131.4 (3)
C6—C7—C14—C15	−52.5 (3)	O2—C27—C28—O1	−59.5 (3)
C9—C7—C14—C15	64.7 (3)	C26—C27—C28—O1	59.7 (3)
C8—C7—C14—C15	−169.2 (2)	O2—C27—C28—C30	61.9 (3)
C6—C7—C14—C12	174.6 (2)	C26—C27—C28—C30	−178.8 (3)
C9—C7—C14—C12	−68.1 (3)	O2—C27—C28—C29	−175.7 (2)
C8—C7—C14—C12	58.0 (3)	C26—C27—C28—C29	−56.4 (3)
C12—C14—C15—C16	−56.1 (3)	C26—C27—O2—C23	25.7 (3)
C7—C14—C15—C16	168.9 (2)	C28—C27—O2—C23	151.5 (2)
C14—C15—C16—O3	−177.17 (19)	C24—C23—O2—C27	87.9 (2)
C14—C15—C16—C17	58.4 (3)	C22—C23—O2—C27	−152.78 (19)
O3—C16—C17—C22	54.6 (3)	C25—C23—O2—C27	−30.1 (2)
C15—C16—C17—C22	175.3 (2)		

### Hydrogen-bond geometry (Å, °)

**Table:**

*D*—H···*A*	*D*—H	H···*A*	*D*···*A*	*D*—H···*A*
O7—H7B···O4	0.84	2.05	2.836 (3)	155
O6—H6C···O5^i^	0.83	2.24	2.791 (3)	124
O7—H7A···O1^ii^	0.84	2.02	2.829 (3)	160
O6—H6D···O3^iii^	0.85	2.04	2.879 (3)	167
O5—H5···O7^iv^	0.82	1.92	2.734 (3)	174
O4—H4···O6^v^	0.82	2.00	2.795 (3)	164
O3—H3···O2	0.82	1.82	2.627 (2)	170
O1—H1···O3	0.82	2.14	2.938 (3)	164

Symmetry codes: (i) *x*−1/2, −*y*+3/2, −*z*; (ii) −*x*+1, *y*+1/2, −*z*+1/2; (iii) *x*−1, *y*+1, *z*; (iv) −*x*+3/2, −*y*+1, *z*−1/2; (v) −*x*+1, *y*−1/2, −*z*+1/2.

## References

[bb1] Bruker (1997). *SMART* and *SAINT* Bruker AXS Inc., Madison, Wisconsin, USA.

[bb2] Iljin, S. G., Mallnovskaya, G. V., Uvarova, N. I., Elyakov, G. B., Antipin, M. Yu & Struchkov, Yu. T. (1982). *Tetrahedron Lett.* **23**, 5067–5070.

[bb3] Sheldrick, G. M. (2008). *Acta Cryst.* A**64**, 112–122. 10.1107/S0108767307043930 18156677

[bb4] Shi, Q., Hen, K., Jioka, T. & Mhiwada, Y. (1992). *J. Nat. Prod.* **55**, 1488–1497.

[bb5] Shibata, S., Tanaka, L., Shoji, L. & Saito, H. (1985). *Econ. Med. Res.* pp. 217–284.

[bb6] Takano, K., Midori, T., Eiichiro, I. & Teruo, M. (1999). *Cancer Lett.* **143**, 11–16.

[bb7] Yu, C., Fu, H., Yu, X., Han, B. & Zhu, M. (2007). *Arzneimittelforschung*, **111**, 568–572.

